# Reply to Li et al., “Kappa Values in Testing the Concordance: Comments on a Recent Article about Nasopharyngeal Swabs for SARS-CoV-2 Detection”

**DOI:** 10.1128/spectrum.03418-22

**Published:** 2022-11-01

**Authors:** Cody Callahan, Sarah Ditelberg, Sanjucta Dutta, Nancy Littlehale, Annie Cheng, Kristin Kupczewski, Danielle McVay, Stefan Riedel, James E. Kirby, Ramy Arnaout

**Affiliations:** a Department of Radiology, Beth Israel Deaconess Medical Center, Boston, Massachusetts, USA; b Department of Pathology, Beth Israel Deaconess Medical Center, Boston, Massachusetts, USA; c Beth Israel Deaconess Medical Center, Boston, Massachusetts, USA; d Division of Clinical Informatics, Department of Medicine, Beth Israel Deaconess Medical Center, Boston, Massachusetts, USA; e Harvard Medical School, Boston, Massachusetts, USA; University of Cincinnati

## REPLY

In a letter regarding our paper (1), Li et al. recalculated Cohen’s *κ* values from the 2 × 2 tables from our Fig. 4 and obtained slightly different values (2). Li et al. pointed out that none of these differences affect the conclusions of our paper but asked the reason for the differences.

The reason for the difference is that, in our study, we used a different cutoff for positive values creating the 2 × 2 tables than was used for calculating the *κ* values. There is argument in the field as to whether cycle threshold (*C_T_*) values significantly above the limit of detection (LOD) are potential false positives. Therefore, to be conservative and not overestimate sensitivity, we used the LOD as a cutoff for the 2 × 2 tables (to reduce the likelihood of false positives); however, for the *κ* calculations, we used *C_T_* values without cutoffs, to reflect qualitative data as reported out by our testing platform according to Emergency Use Authorization (EUA) approval as a basis for calculated agreement. It would have been clearer to have done the same thing for both calculations (although as Li et al. pointed out, the difference does not affect our paper’s conclusions).

**FIG 1:**
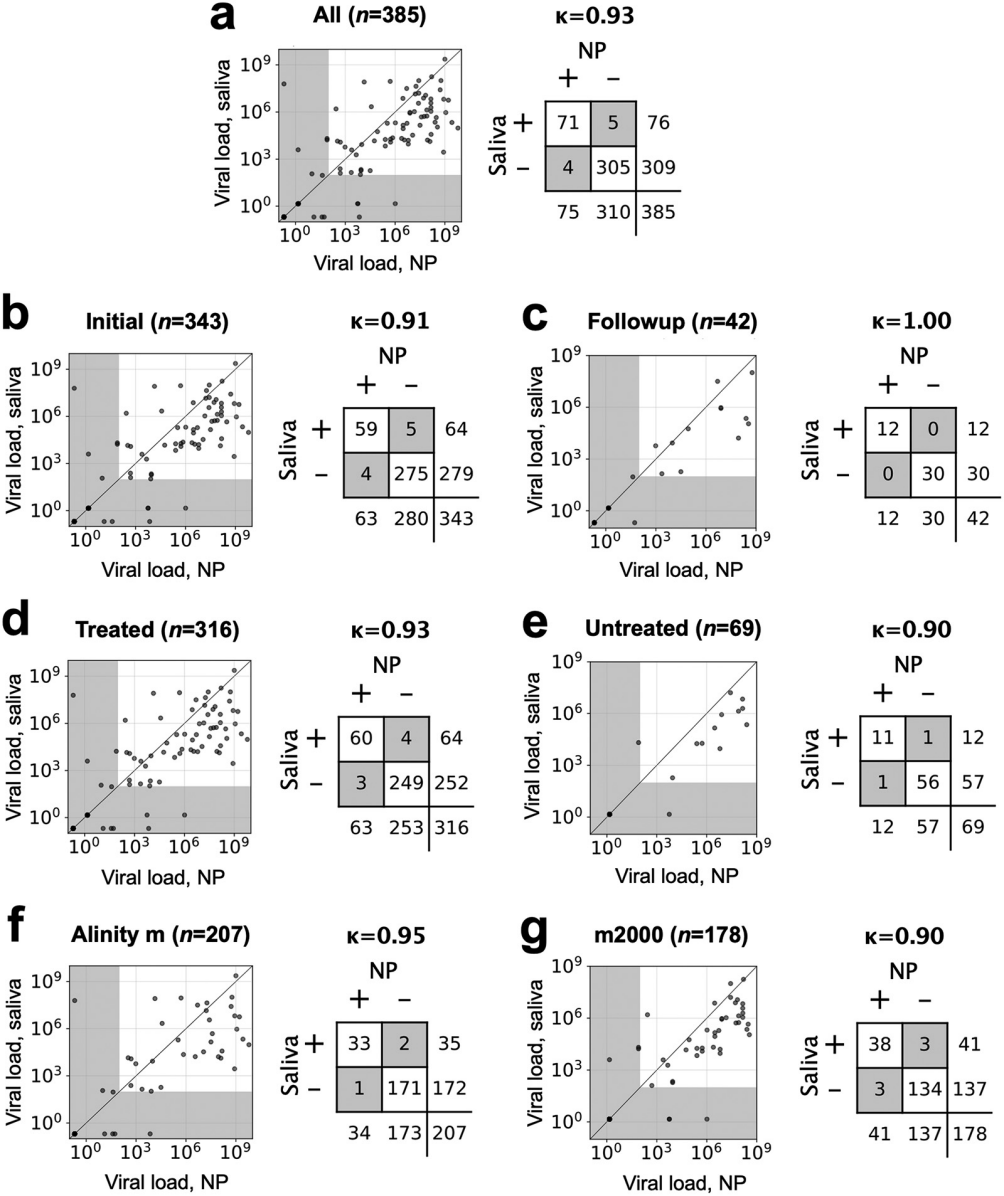
Viral load in saliva versus NP swab samples, Cohen’s kappa (*κ*) concordance values, and contingency tables for overall study (a), subjects presenting for initial presentation (within 5 days of first COVID-19 RT-PCR test) (b), subjects presenting for follow-up testing (c), samples treated with GITC (guanidinium isothiocyanate) transport buffer as a preservative after receipt at the central laboratory (d), untreated samples (e), samples run on the Alinity m platform (f), and samples run on the m2000 (g). Diagonal lines in scatterplots, 1:1. Gray-shaded areas in the scatterplots are below the LOD (100 copies/mL). Gray-shaded cells in the contingency tables highlight discordant results. In all cases, the LOD was used as a cutoff for positive versus negative.

Figure 1 is an update of Fig. 4 from our paper (1), making all values consistent by using LOD cutoffs throughout. Our *κ* values (obtained using Python’s scikit-learn package) are now identical to those of Li et al. (obtained using the commercial software SPSS) except for panel g, where we obtain 0.90 compared to Li et al.’s 0.91. We suspect this discrepancy is due to rounding: the value to multiple decimal places is *κ* = 0.9049…, which rounds down to 0.90. Possibly SPSS returned only three decimal places—0.905—which would round up to 0.91. This difference is unimportant.

We believe the letter by Li et al. and our response highlight the value of both critical feedback and transparency (3, 4), especially the advantage of our having provided the entirety of our data set to facilitate reanalysis. We made all data and code available on GitHub when the original paper was published and have revised the Python notebook there that produces our results in light of Li et al.’s letter. We favor this practice, as well as the use of open-source software—here, Python instead of proprietary software like SPSS, which is less available and therefore harder to vet—in the interest of open science.

We thank Li et al. again for their careful reanalysis of our data.

Ramy Arnaout, on behalf of the authors.
